# Examining Problem Behaviors and Social Skills in Preschoolers Exhibiting Early Signs of Learning Disabilities

**DOI:** 10.3390/bs15081087

**Published:** 2025-08-12

**Authors:** Yalcin Aydin, Ibrahim Halil Diken

**Affiliations:** 1 Akademi Disleksi, 16230 Nilüfer, Türkiye; yalcinaydin@akademidisleksi.com; 2 Research Institute for Individuals with Developmental Disabilities, Anadolu University, 26470 Tepebaşı, Türkiye

**Keywords:** learning disability, early signs, problem behavior, social skills, preschoolers

## Abstract

This study examines problem behaviors and social skills among children aged 4–6 who are exhibiting early signs of learning disabilities. The data were collected via online surveys from 153 parents of children in private preschools in Bursa in Türkiye. A quantitative research design coupled with the correlational method was employed. The results indicate that exhibiting higher levels of early signs of learning disabilities correlates with increased problem behaviors and reduced social skills, particularly in regard to social communication and interaction. Children exhibiting lower levels of early signs of learning disabilities show stronger social skills and fewer behavioral issues. These findings highlight the need for early intervention, targeted social skills training, and behavior regulation strategies to support children exhibiting early signs of learning disabilities.

## 1. Introduction

Learning disabilities (LDs) are defined as neurologically based processing disorders, which can interfere with learning basic skills, such as reading, writing, or math, as well as higher-level skills, such as organization, time planning, abstract reasoning, long or short-term memory, and attention (American Psychiatric Association, 2013). The DSM-5 defines specific learning disabilities as persistent difficulties in key academic areas, despite the provision of interventions targeting those difficulties. Importantly, LDs are not a reflection of intelligence levels, sensory deficits, or socio-economic deprivation, but rather result from intrinsic differences in the brain’s processing of information. Children with LDs often appear in classrooms across all educational levels, yet the early identification of those exhibiting signs remains inconsistent, especially in early childhood. This issue is particularly problematic given the widespread agreement that early intervention is critical for mitigating the long-term effects of LDs (Guralnick, 2011). Indeed, the foundation for learning, both academic and social, is laid during the early childhood period, and delays in any domain during these formative years can lead to compounding difficulties later on.

Children with LDs may struggle with attention regulation, impulse control, and frustration tolerance, leading to negative peer interactions and strained teacher relationships. These problem behaviors, in turn, exacerbate academic difficulties, forming a cycle of negative outcomes that can persist into adolescence and adulthood. Moreover, children exhibiting early signs of LDs often demonstrate lower levels of executive functioning and cognitive flexibility, skills that are crucial for adapting to social norms, negotiating conflicts, and maintaining relationships (Bull & Espy, 2006). As such, these children may struggle with understanding social cues, managing emotions in group settings, or following multi-step directions, further alienating them from their peers and educators alike.

Social skills are a set of learned behaviors that facilitate interaction and communication with others. These include both verbal and non-verbal behaviors, such as initiating conversations, cooperating with others, expressing needs appropriately, and interpreting social cues (Gresham & Elliott, 1990). Social competence is considered a vital aspect of school readiness and is predictive of later academic success, emotional well-being, and life satisfaction (Denham et al., 2003). During the preschool years, children begin to master the basic foundations of social interaction. They learn to cooperate, take turns, resolve conflicts, and empathize with others, skills that are not only essential for personal development, but also for academic participation and school success (Ladd et al., 1996). However, when these skills are underdeveloped, children are at increased risk of peer rejection, social isolation, and behavioral problems, which can significantly hinder their academic progress and psychological development.

Behavioral problems and social skill deficits often have a reciprocal relationship. Children who lack social skills are more likely to engage in inappropriate behaviors, which in turn further impede their social development. For example, a child who has difficulty sharing or turn taking may resort to aggression when frustrated, leading to negative responses from their peers and adults, which reinforces maladaptive behavior patterns (Walker et al., 1991). Research by McClelland et al. (2000) suggests that poor self-regulation and social competence in preschool children are strongly associated with increased problem behaviors and lower academic performance in elementary school. Similarly, Fantuzzo et al. (2005) found that preschool children with poor social adjustment exhibited higher levels of aggressive behavior and were more likely to require special education services later on.

Children exhibiting early signs of learning disabilities (LDs) differ from those with confirmed diagnoses in that they exhibit warning signs of learning challenges, but may not yet meet diagnostic criteria. These early signs can be identified through a combination of developmental screenings, teacher observations, and assessments of early academic and behavioral functioning. Such children often display emerging difficulties in language acquisition, attention control, and early literacy skills, signs strongly associated with future learning disabilities. The concept of the “identification of early signs” is critical because it allows for early interventions before academic failure becomes entrenched. Studies suggest that targeted interventions at the preschool stage can dramatically alter developmental trajectories for those exhibiting early signs of LDs (Torgesen, 2000).

Much of the existing literature on LDs focuses on school-aged children, with limited attention paid to preschool-aged children who are exhibiting early signs of LDs. Even fewer studies simultaneously investigate both social skills and problem behaviors in this group, despite mounting evidence that these domains are interrelated and critical for school readiness. The National Early Childhood Technical Assistance Center (2009) emphasizes the importance of including social–emotional development as a core component of early intervention services, yet empirical studies focusing on this integration remain limited. Moreover, when problem behaviors and social skills are studied, they are often examined in isolation rather than in conjunction with early signs of LDs. This siloed approach fails to capture the complex interactions between these domains and underestimates the cumulative burden faced by children exhibiting early signs of LDs. Understanding these interactions is essential for developing holistic intervention models that address both academic and behavioral needs.

Building on this theoretical foundation, the current study focuses on two key developmental domains, social skills and problem behaviors, as they relate to early signs of LDs. According to Bronfenbrenner’s (1979) model, difficulties in regard to proximal processes, such as peer and teacher interactions, can disrupt developmental trajectories, particularly in educational contexts. Vygotsky’s (1978) theory supports this view by emphasizing how social engagement mediates cognitive development; thus, children who struggle with behavioral regulation or social participation may experience reduced access to learning opportunities. These perspectives guided the selection of variables in this study, with problem behaviors and social skills conceptualized as both potential outcomes of developmental risk and mediators of learning engagement. Accordingly, the hypotheses suggest that increased problem behaviors and reduced social skills are associated with a heightened risk of learning difficulties, and that these variables may also serve as reliable predictors of behavioral problems.

While Bronfenbrenner’s and Vygotsky’s theories provide an overarching lens for understanding the interaction between social and cognitive development, more recent pedagogical frameworks offer targeted explanations of how behavioral and social competencies directly influence learning. For example, self-regulation and executive function theories (Blair & Raver, 2015) highlight how children’s ability to manage their attention and behavior predicts their learning trajectories. Similarly, social–emotional learning (SEL) research (Denham, 2006; Jones et al., 2015) has demonstrated that social competence supports academic engagement and classroom success. These models provide a more proximal rationale for the inclusion of problem behaviors and social skills as central variables in the current study.

Understanding the interplay between problem behaviors and social skills in children exhibiting early signs of LDs has significant implications for early childhood education, mental health services, and policy development. Teachers, school psychologists, and intervention specialists require comprehensive, empirically grounded frameworks to design and implement effective support systems. This study aims to provide such a framework by identifying key behavioral markers associated with early signs of LDs and their implications for social development. They may also inform curriculum design in preschool programs by integrating social–emotional learning modules that specifically address the needs of children exhibiting early signs of academic difficulty.

### The Purpose

The purpose of this study is to examine the levels of problem behaviors and social skills in children aged 4–6 who are exhibiting early signs of LDs. In line with this objective, the following research questions and hypotheses are addressed:

1. What are the levels and scores in terms of problem behaviors, social skills, and early signs of LDs among the participating children?
2. Is there a significant relationship between the level of early signs of LDs and the levels of problem behaviors and social skills in children exhibiting early signs of LDs?

**Hypothesis** **1.** *Exhibiting higher levels of early signs of LDs will be positively correlated with higher levels of problem behaviors and negatively correlated with social skills.*

3. Are there significant differences in problem behavior and social skill scores for children exhibiting different levels of early signs of LDs?

**Hypothesis** **2.**
*Children exhibiting higher levels of early signs of LDs will have significantly higher problem behavior scores and significantly lower social skills scores compared to children exhibiting lower levels of early signs of LDs.*

4. Does the level of early signs of LDs and social skill scores significantly predict problem behaviors?

**Hypothesis** **3.**
*Both exhibiting higher levels of early signs of LDs and lower social skills scores will significantly predict higher levels of problem behaviors.*

## 2. Method

### 2.1. Research Model

In this study, which aims to examine the problem behavior and social skills levels of children exhibiting early signs of LDs, the correlational method was used. The correlational method aims to determine the existence and/or degree of change between two or more variables (Karasar, 2012).

### 2.2. Participants and Data Collection

The study group in this research consists of 153 parents of children between the ages of 4–6 years (48–72 months) exhibiting early signs of LDs at private preschools in Bursa. The criterion sampling method, which is one of the purposive sampling methods, was used to determine the study group. The criterion sampling method involves the creation of a participant group involving people, objects, events, and situations with characteristics related to the research problem (Büyüköztürk, 2014). Before the data collection process was carried out in regard to the research, an application was made to Anadolu University Social and Human Sciences Scientific Research and Publication Ethics Committee for “Research Ethics Committee Permission” and the relevant permission (Protocol No: 721805) was obtained. Private preschools in Bursa were contacted, and their consent was obtained for their voluntary participation in the study. Since we assume that preschool teachers know their students well, preschool teachers were informed about the early signs of LDs based on the Screening Scale for Early Symptoms of Learning Disabilities (SSESLD) and were asked to nominate suitable children. Then, the parents of the children nominated by the teachers were asked to provide consent and sign the consent form for the study. The research data were collected online via Google Forms, with a link sent to the participants who had provided their consent.

Out of 153 children, 73 (47.7%) of the children were boys, 80 (52.3%) were girls. The mean age of the children was 68.9 months (SD = 3.53, Min = 51, Max = 72), while the mean age of the parents was 33.6 years (SD = 4.59, Min = 25, Max = 51). A total of 137 (89.5%) mothers and 16 (10.5%) fathers participated in the study. The mean duration of school attendance was 6.37 months (SD = 4.55, Min = 1, Max = 24).

### 2.3. Data Collection Tools

The following data collection tools were used in the study:

Demographic Information Forms. A form was added at the beginning of the data collection tool to obtain information on the parents and the child’s age, gender, and how long the child had been receiving education.

Screening Scale for Early Symptoms of Learning Disabilities (SSESLD; Okur & Aksoy, 2024). The SSESLD is a scale designed to assess children aged 4–6 who exhibit early signs of LDs, based on their parents’ observations. The SSESLD consists of four subscales, containing 52 items, that include observable and measurable behaviors associated with learning disabilities. The items included in the SSESLD were developed based on DSM-5, developmental, cognitive, and phonological approaches. The subscales are language development and communication, cognitive skills, psychomotor development skills, and social–emotional skills. The first subscale, language development and communication, consists of 14 items. The second subscale, cognitive skills, consists of 19 items. The third subscale, psychomotor development skills, consists of 13 items. The fourth subscale, social–emotional skills, consists of 7 items. Scale items are scored on a scale of 1 to 5 (‘1’—completely disagree–‘5’—completely agree). A total of 1429 parents of children with learning difficulties participated in the study. The scale showed high reliability, with a Cronbach’s alpha coefficient of 0.95 in terms of internal consistency, and the content validity index was found to be 0.92. It was determined that the scale explains 48.99% of the total variance, according to its four-factor structure. The fit indices obtained in the confirmatory factor analysis were CFI = 0.97, SRMR = 0.079, NNFI = 0.96, and RMSEA = 0.068, indicating that the model fits the data well. The scores are converted into standard scores, and the standard scores are converted into an LD index, and, as the index score increases, the occurrence of early signs of LDs is interpreted as “Very Low”, “Mild”, “Moderate”, or “High”.

Preschool and Kindergarten Behavior Scale-2 (PKBS-2; Fazlıoğlu et al., 2011). The Preschool and Kindergarten Behavior Scale (PKBS-2) was developed by Merrell (2003) and adapted into Turkish by Fazlıoğlu et al. (2011), involving 201 children (102 girls and 99 boys) aged 3–6 years. In the Turkish adaptation study involving the Preschool and Kindergarten Behavior Scale-2 (PKBS-2), it was determined that the factor structure of the 76-item scale (Problem Behavior Scale 42 items, Social Skills Scale 34 items) was preserved in Turkish culture. The Social Skills Scale (SSS) has three subscales with the titles “Social Cooperation, Social Interaction and Social Independence”, while the Problem Behavior Scale (PBS) has two subscales with the titles “Internalizing Behaviors and Externalizing Behaviors”. The items in the scale are scored as “0 (Never), 1 (Rarely), 2 (Sometimes), and 3 (Frequently)” and a high social skills score briefly defines an excellent level of social adaptation, while a high problem behavior score briefly defines a high level of problem behavior.

### 2.4. Data Analysis

After the data were collected, they were visually checked, and the prerequisites were tested before the relevant analyses. Two criteria were examined in order to ensure the normal distribution characteristics of the data. These were the Shapiro–Wilk and Kolmogorov–Smirnov significance values (*p* > 0.05), and the value obtained by dividing the Skewness and Kurtosis values by the standard error values should be between −1.96 and +1.96 (Tabachnick & Fidell, 2007). While the Kolmogorov–Smirnov test was used for variables with more than 30 observations, the Shapiro–Wilk significance values were taken into consideration when the number of observations was below 30. In addition, missing values were checked and assigned using the average method (Pallant, 2011). The analyses showed that the data were normally distributed and, therefore, parametric analyses were carried out. In regard to the comparison analyses, the Bonferroni correction was used to avoid Type 1 errors, which cause a non-significant difference to appear significant (Pallant, 2011). Cohen’s effect size interpretation (1988) was used in regard to the η^2^ values obtained. Accordingly, “0.1” was considered as a small effect, “0.3” as a medium effect, and “0.5 and above” as a large effect.

## 3. Results

### 3.1. Results Related to Levels of Early Signs of LDs, Problem Behaviors, and Social Skills Levels of Participant Children

The levels of early signs of LDs, problem behaviors, and social skills of the participant children were examined using descriptive statistics. According to the SSESLD, 10.5% (n = 16) of the participant children were categorized as “Low level occurrence of early signs of LDs”, 75.2% (n = 115) as “Moderate level occurrence of early signs of LDs”, and 14.4% (n = 22) as “High level occurrence of early signs of LDs”.

Social skill levels were examined on the basis of social cooperation, social interaction, social independence, and the total social skills score. By considering the minimum and maximum values of these scores, the distribution and variability in children’s social skill levels can be evaluated more clearly. Social cooperation reflects children’s ability to collaborate with others and participate in group activities. The average score (X¯ = 27.20, SD = 5.42) was quite close to the highest score of 36. This shows that the children generally have good social cooperation skills. However, since the standard deviation was 5.42, it can be said that there was a certain amount of variation among the children. Social interaction measures children’s ability to communicate and share with their peers and adults and to be active in social settings. The average score (X¯ = 26.61, SD = 4.78) was quite close to the highest score of 33. This shows that the majority of children have strong social interaction skills. However, the standard deviation of 4.78 indicates that children’s social interaction levels were partially consistent with each other, but that individual differences cannot be ignored. Social independence measures children’s ability to act independently, make decisions on their own, and be individually active in social settings. The mean score (X¯ = 25.61, SD = 4.69) was quite close to the highest score of 33, which indicates that the children generally have high social independence skills. However, the standard deviation of 4.69 indicates that there were individual differences, but that the social independence levels were relatively homogeneously distributed. The total social skills score was obtained by combining social cooperation, interaction, and independence scores. The mean score (X¯ = 79.41, SD = 13.44) shows that the children have a good level of social skills, in general. The standard deviation of 13.44 indicates that the difference between individuals was larger than for the other subscales and that there were significant differences in the social skill levels among the children. 

Within the scope of the problem behavior scale, three main variables were addressed: internalizing behaviors, externalizing behaviors, and the total problem behavior score. The internalizing score measures children’s behaviors such as shyness, social withdrawal, passivity, and introversion. The mean score (X¯ = 15.26, SD = 6.35) was quite low compared to the maximum score for the scale (81), indicating that children’s tendency towards introversion was low, in general. On the other hand, the standard deviation of 6.35 indicates that there were individual differences among the children. The externalizing score measures externalizing problem behaviors such as aggression, anger, hyperactivity, and inappropriate social behaviors. The mean score (X¯ = 26.95, SD = 12.11) was quite close to the maximum score of 45, which shows that children tend to exhibit externalizing problem behaviors in general. The standard deviation of 12.11 shows that there was a significant difference between individuals. The total problem behavior score determines the general problem behavior level of children by evaluating both internalizing and externalizing problem behaviors together. The mean score (X¯ = 42.21, SD = 17.15) was approximately one third of the maximum score of 126, which indicates that the children’s problem behavior levels were generally moderate.

The children’s language development and communication skills, cognitive skills, psychomotor skills, and social–emotional skills were examined within the scope of the Screening Scale for the Early Symptoms of Learning Disabilities (SSESLD). Language development and communication skills measures the children’s speaking, listening, understanding, and communication skills. The mean score (X¯ = 28.43, SD = 10.03) was low compared to the maximum score of 70, indicating that the children generally have moderate skills in this area. The standard deviation of 10.03 reveals that there were significant individual differences among the children. This suggests that some children may have much weaker communication skills than their peers. Cognitive skills assess the children’s thinking, problem solving, memory, attention, and learning processes. The mean score (X̄ = 46.60, SD = 11.07) was approximately half of the maximum score of 90, indicating that the children were generally at an intermediate level in terms of their cognitive skills. However, the standard deviation of 11.07 reveals that there were significant individual differences in the cognitive abilities among the children. This suggests that although some children have age-appropriate cognitive skills, while some children have significant problems in regard to the learning process. Psychomotor skills assess children’s fine and gross motor skills, coordination abilities, and physical movements. The mean score (X¯ = 28.22, SD = 7.74) was relatively low compared to the maximum score of 65, indicating that the children may need support in this area. The standard deviation of 7.74 indicates that there were significant individual differences in the motor skills among the children. Social emotional skills measures children’s ability to establish social relationships, manage their emotional reactions, and express themselves. The mean score (X¯ = 15.40, SD = 5.72) was below the maximum score of 35, which indicates that the children may need more support in this area. The standard deviation of 5.72 indicates that there were differences in the social and emotional skill levels among the children. These differences suggest that some children have more difficulties in regard to their social and emotional development compared to their peers. The total score of the SSESLD evaluates children’s overall performance in all the developmental domains (language, cognitive, psychomotor, social–emotional). The mean score (X¯ = 118.65, SD = 24.57) was approximately half of the maximum score of 260, indicating that the general developmental level of the children was at a moderate level. The standard deviation of 24.57 indicates that there were large developmental differences between the children. 

### 3.2. Results on the Relationships Between the Total Scores of Participant Children’s Early Signs of LDs, Problem Behaviors, and Social Skills Scores

The Pearson correlation coefficients and significance levels for the total scores of the children’s early signs of LDs, problem behaviors (externalizing, internalizing, and total problem behavior score), and social skills components (social communication, social interaction, social independence, and total social skills score) are reported.

When the correlations between the components of the children’s social skills were analyzed, there was a strong and positive relationship between social communication and social interaction (r = 0.768, *p* < 0.001). This finding suggests that children with a high level of social communication skills also tend to be successful in regard to social interaction. Social independence was positively correlated with both social communication (r = 0.673, *p* < 0.001) and social interaction (r = 0.728, *p* < 0.001). This finding suggests that children with developed social communication and interaction skills tend to be more socially independent. Very strong positive correlations were found between the total social skills score and all of the social skills components (social communication r = 0.911, social interaction r = 0.919, social independence r = 0.879, *p* < 0.001). This result shows that social skills are complementary constructs, and that all of the skills determine the child’s overall level of social competence. When the correlations between problem behaviors and social skills were examined, it was seen that externalizing behaviors were negatively correlated with social skills components (social communication r = −0.475, social interaction r = −0.247, social independence r = −0.246, social skills total r = −0.365, *p* < 0.05). These findings suggest that children who exhibit extroverted problem behaviors, such as aggression and hyperactivity, may have lower levels of social skills. Internalizing behaviors were negatively related to social skills (social communication r = −0.308, social interaction r = −0.171, social interaction r = −0.171, social independence r = −0.218, social skills total r = −0.261, *p* < 0.05). Children with lower social interaction scores tended to show introversion and social inhibition. The total problem behavior score was negatively correlated with all of the social skills components (r = −0.449 to −0.354, *p* < 0.05). This finding suggests that children with a high level of problem behaviors have low social skills and may have negative social interaction experiences.

When the correlations between early signs of LDs (SSESLD) and other variables were analyzed, the total score of the SSESLD was negatively correlated with social skills components (r = −0.369 to −0.396, *p* < 0.001). This finding suggests that children exhibiting higher levels of early signs of LDs tend to score lower in regard to their social skills. The total score of the SSESLD was positively correlated with the components of problem behaviors (externalizing r = 0.341, internalizing r = 0.347, total problem behavior score r = 0.369, *p* < 0.001). This finding suggests that children exhibiting higher levels of early signs of LDs may be more likely to exhibit problem behaviors.

### 3.3. Results Related to the Difference Between Children’s Problem Behaviors and Social Skills Total Scores According to the Levels of Early Signs of LDs

In this study, the differences between children’s problem behaviors and social skills scores according to the levels of early signs of LDs (moderate, mild, and very low) were examined. The data were analyzed using descriptive statistics, a one-way analysis of variance (Welch’s ANOVA), and the Games–Howell post hoc test.

Table 1 shows the mean and standard deviation of the social skills and problem behavior scores of the children with the levels of early signs of LDs (moderate, mild, very low). In general, as the level of early signs of LDs decreases, the social skills scores increase, and the problem behavior scores decrease. The social communication, social interaction, social independence, and total social skills scores increase as the level of early signs of LDs decrease. For example, the total social skills score was calculated as 69.9 in the moderate group, 79.3 in the mild group, and 86.9 in the very low group. This indicates that children with a low level of early signs of LDs have stronger social skills.

The externalizing, internalizing, and total problem behavior scores increased as the level of early signs of LDs increased. For example, the total problem behavior score was 52.5 in the moderate group, 43.3 in the mild group, and 29.1 in the very low group. Similarly, the externalizing and internalizing scores were higher in children with a high level of early signs of LDs. This indicates that children exhibiting a high level of early signs of LDs exhibit more problem behaviors and that these children are more prone to externalizing (aggression, hyperactivity) and internalizing (shyness, introversion) problem behaviors.

When Table 2 is examined, the results of the one-way analysis of variance (Welch’s ANOVA) show that there is a statistically significant difference between the level of early signs of LDs, social skills, and problem behaviors scores (*p* < 0.007, Bonferroni correction applied). The highest values in terms of the effect size (Eta Square, η^2^) were calculated for the total problem behavior score (η^2^ = 0.220) and internalizing behavior (η^2^ = 0.209). In terms of the social skill levels in particular, the most substantial difference was found for social communication (η^2^ = 0.185) and the total social skill score (η^2^ = 0.186). These findings reveal that children exhibiting higher levels of early signs of LDs have significant deficits in their social communication and interaction skills. At the same time, the differences in the externalizing (η^2^ = 0.197) and internalizing (η^2^ = 0.209) behavior scores indicate that children exhibiting a higher level of early signs of LDs are more likely to have social–emotional problems.

When the results of the post hoc analyses (Games–Howell test) were analyzed, it was observed from the data presented in Table 3 and Table 4 that children exhibiting a “very low” level of early signs of LDs were significantly different from the other groups. In terms of the total problem behavior score, the difference between the moderate and very low group (*p* < 0.001) and the difference between the mild and very low group (*p* < 0.003) were significant. Similarly, in terms of internalizing and externalizing behavior scores, children exhibiting a higher level of early signs of LDs were found to be significantly different from the other groups. In terms of the internalizing behavior score, the difference between the moderate and very low group (*p* < 0.001) and the mild and very low group (*p* < 0.025) were significant. In terms of external orientation, significant differences were found between the moderate and very low group (*p* < 0.001) and between the mild and very low group (*p* < 0.003). However, no significant differences were observed between the moderate and mild group in regard to some of the variables (for example, *p* = 0.077 for the total problem behavior score). These findings clearly reveal that children exhibiting a higher level of early signs of LDs are more likely to show inadequacy in terms of their social skills and exhibit problem behaviors. In particular, children exhibiting a lower level of early signs of LDs have the highest scores in terms of their social skills and the lowest level of problem behaviors.

### 3.4. Results of Regression Analysis on the Prediction of Problem Behaviors According to the Level of Early Signs of LDs and Social Skills Score

The predictive power of students’ social skill levels and the level of early signs of LDs variables for problem behaviors was analyzed using a three-stage hierarchical regression analysis and the results are provided in Table 5.

In regard to the first model, the level of early signs of LDs was included as an independent variable. The analysis showed that this variable was positive and significant in terms of the total problem behavior score and explained approximately 13.6% of the total variance (R^2^ = 0.136, *p* < 0.001, B = 0.258, Beta = 0.369). In regard to the second model, the total social skills score was added to the analysis and a significant increase of approximately 5.2% was observed in the explanatory power of the model (ΔR^2^ = 0.052, *p* < 0.001). In this model, the level of early signs of LDs (B = 0.189, Beta = 0.271, *p* = 0.001) and the total social skills score (B = −0.315, Beta = −0.247, *p* = 0.002) variables were found to have significant predictive effects on the total problem behavior score. The results obtained from the first two models show that the level of early signs of LDs variable predicts the total problem behavior score positively, while the total social skills score predicts the total problem behavior score negatively. It was determined that the two variables explained approximately 18.8% of the variance related to problem behaviors in total. In the third model, the interaction between early signs of learning difficulties and social skills was tested to determine whether their combined effect contributed to the prediction of social skills levels. However, this interaction did not yield a significant contribution to the model (ΔR^2^ = 0.002, *p* > 0.05).

## 4. Discussion

This study aimed to examine the levels of problem behaviors and social skills in children aged 4 to 6 who show early signs of LDs. The data collected within the scope of this research were analyzed, and several significant findings were obtained.

The analyses revealed that children exhibiting higher levels of early signs of LDs show underdeveloped language and communication skills, including difficulties in speaking, listening, understanding, and engaging in communication. These findings suggest that some children possess significantly weaker communication abilities compared to their peers. According to Elksnin and Elksnin (2004), children exhibiting higher levels of early signs of LDs often experience low self-confidence due to poor language skills, difficulty in understanding others’ emotions through speech and comprehension, repeated academic failures, and educational isolation. Gadeyne et al. (2004) also emphasize that problem behaviors often stem from communication problems caused by weak language skills. The literature indicates that the presence of poor language abilities impairs social interaction among children exhibiting signs of LDs, thereby exacerbating their communication difficulties (Kavale & Forness, 1996; Remington et al., 2007; Webber & Plotts, 2008).

The evaluations of cognitive skills, including thinking, problem solving, memory, attention, and learning processes, demonstrated that while some children exhibit age-appropriate cognitive abilities, others encounter noticeable difficulties. The findings suggest that children exhibiting higher levels of early signs of LDs struggle to interpret emotions expressed through body language, compared to their typically developing peers. Kılıç-Tülü and Ergül (2016) similarly found that children exhibiting signs of LDs perform significantly lower in terms of emotion recognition and have greater difficulty interpreting body language. These challenges stem from sensory processing issues that affect learning. Bloom and Heath (2010) reported that children exhibiting signs of LDs exhibit lower performance in recognizing and interpreting emotional facial expressions.

When analyzing the relationships between components of social skills, a strong and positive correlation was found between social communication and social interaction. This suggests that children who possess strong social communication abilities are also more likely to engage successfully in social interactions, a finding consistent that is with previous studies (Agaliotis & Kalyva, 2008; Ceylan & Yiğitalp, 2018; Değirmenci, 2022; Tokuşlu, 2022). Additionally, social independence positively correlates with both social communication and social interaction, indicating that children with more developed language and interaction skills tend to demonstrate higher levels of social autonomy. Conversely, limited language proficiency among children at risk of learning disabilities negatively affects their social communication and interaction skills. Hence, children whose language abilities support their independence also benefit from enhanced communication and interaction skills (Kavale & Forness, 1996; Webber & Plotts, 2008). Further analyses indicated very strong positive correlations between the total social skills score and all of its subcomponents. This supports the notion that social skills function as interdependent constructs, with each contributing to a child’s overall social competence (Agaliotis & Kalyva, 2008; De Munter & Ghesquière, 1999; Özen, 2015). 

This study also explored the relationship between problem behaviors and social skills. Externalizing behaviors, such as aggression and hyperactivity, were negatively correlated with all of the social skill subcomponents. This suggests that children exhibiting these behaviors tend to have lower social skill levels. Similarly, internalizing behaviors, such as social withdrawal and shyness, also showed negative correlations with social skills. Overall, children with higher levels of problem behavior tend to demonstrate weaker social skills and experience negative social interactions. Heiervang et al. (2001) found that children at risk of learning disabilities display more behavioral problems compared to their peers, especially in relation to externalizing, internalizing, and attention-related issues. D’Amico and Guastaferro (2017) noted that both externalizing (e.g., aggression, hyperactivity) and internalizing (e.g., withdrawal, shyness) behaviors are significant predictors of problem behavior in children with learning disabilities. Similar findings were reported by Milligan et al. (2016), Sanford and Horner (2013), and others, although Raimundo et al. (2013) found that social skill deficits did not significantly affect problem behaviors.

The correlational analysis of early signs of LDs using the SSESLD showed a negative correlation between the SSESLD total scores and social skill components, suggesting that higher levels of early signs of LDs are associated with lower social skill levels. Conversely, a positive correlation was found between the SSESLD scores and problem behavior components, indicating that children exhibiting higher levels of early signs of LDs are more likely to exhibit problem behaviors. In general, as the level of early signs of LD risk decreases, social skills improve, and problem behaviors decline. These findings are consistent with the literature (Ezmeci et al., 2022; Gresham et al., 2001; Kılıç & Güngör Aytar, 2017; Milligan et al., 2016; Tokuşlu, 2022; Vural, 2009). Similarly, numerous studies confirm that children exhibiting early signs of LDs exhibit elevated levels of problem behaviors (Aksakal, 2020; Aksoy & Baran, 2020; Çayır & Eid, 2010; Değirmenci, 2022; Kavale & Forness, 1996; Manassis & Young, 2000). 

In conclusion, the findings from this study reveal that the level of early signs of LDs significantly impacts both children’s social skills and problem behaviors. Children exhibiting higher levels of early signs of LDs demonstrate marked deficits in social communication and interaction, as well as a greater likelihood of exhibiting problem behaviors. In contrast, children exhibiting lower levels of early signs of LDs attain the highest social skill scores and the lowest levels of problem behaviors. These findings highlight the urgent need to develop early intervention programs targeting children exhibiting early signs of LDs, as well as the need to enhance social skills education and implement structured behavior regulation programs. The literature supports the finding that social skills training can effectively reduce problem behaviors in this population (Duering & Petermann, 2011; Ezmeci et al., 2022; Kılıç & Güngör Aytar, 2017; Most & Greenbank, 2000; Özbey, 2009). Educational support programs not only reduce, but may also neutralize, problematic behaviors (Cen & Aytaç, 2017). Studies have shown that such programs improve children’s social skills, whereas the absence of interventions often leads to an increase in problem behaviors (Aykır & Çiftçi-Tekinarslan, 2012; Ceylan & Yiğitalp, 2018; McCoy et al., 2012; Pijl et al., 2013).

While this study’s overall contribution may be considered moderate within the broader literature, it nonetheless adds to the growing body of empirical research underscoring the need for early identification and intervention in regard to children showing early signs of LDs. As the evidence base continues to expand, so does the justification for allocating more resources to support children exhibiting early developmental risks. Research efforts such as this one, particularly those emerging from underrepresented regions, strengthen the global call for accessible, early intervention strategies, thereby promoting more equitable educational and developmental outcomes.

### 4.1. Limitations

While this study provides valuable insights into the problem behaviors and social skills of children exhibiting early signs of LDs, several limitations should be noted. The study was limited to a specific group of 153 children aged 4–6 years, enrolled in private preschools in Bursa. Therefore, the findings may not be generalizable to broader populations, including children attending public institutions or residing in other regions of Türkiye. The use of online survey techniques to gather data from parents may have introduced response bias. Parents’ interpretations and subjective perceptions of their children’s behaviors could have influenced the accuracy of the reported information. The study employed a cross-sectional design, capturing data at a single point in time. This limits the ability to draw causal inferences or examine developmental changes over time. Although the instruments used were reliable and valid, the study relied solely on parent-reported measures without incorporating teacher evaluations or direct observational data, which could have provided a more comprehensive assessment of children’s behaviors and skills. The study focused on teacher-nominated and parent-reported children exhibiting early signs of LDs, but it did not include a diagnostic follow-up or clinical validation to confirm the presence of such disabilities. Future studies should consider longitudinal designs, include more diverse participant groups, and utilize multi-informant assessment methods to deepen the understanding and improve the generalizability of the results.

A potential limitation of this study lies in the conceptual overlap between the predictor measure (SSESLD) and the outcome measures (PKBS-2), particularly regarding social–emotional functioning. While the SSESLD includes a social–emotional subscale, it primarily assesses language, cognitive, and motor development. Moreover, the PKBS-2 was developed independently to capture behavioral manifestations rather than developmental precursors. Nonetheless, we recognize the importance of using multiple sources and informants in future studies to ensure a clearer delineation of predictor and outcome constructs.

### 4.2. Implications for Practice

Although the study was conducted in Türkiye, the patterns of problem behaviors and social skill deficits among preschoolers exhibiting early signs of LDs align with findings from diverse international contexts (Booth-LaForce & Oxford, 2008; Guralnick, 2011). This underscores the universal nature of the challenges faced by this population, regardless of geographic location. The inclusion of non-Western data contributes to a more comprehensive understanding of early developmental risks and emphasizes the need for global investment in early intervention strategies (UNESCO, 2020; World Health Organization, 2012). By expanding the geographical diversity of the research base, studies like this one support advocacy for equitable resource allocation and intervention access worldwide (Black et al., 2017; Engle et al., 2011).

The findings from this study underscore the urgent need for early and integrated educational interventions targeting both social–emotional and behavioral development in children exhibiting early signs of LDs. Given the strong negative correlations between the levels of early signs of LDs and social skills and the positive association with problem behaviors, early childhood educators and intervention specialists should prioritize social–emotional learning (SEL) as a foundational component of preschool curricula. One practical implication is the implementation of targeted social skills training programs in early education settings, particularly for children identified through screening as exhibiting early signs of LDs. These programs should emphasize communication, cooperation, conflict resolution, and emotional regulation, skills found to be deficient in this population. Embedding SEL activities into daily routines can promote social competence and reduce the likelihood of problem behaviors escalation.

Moreover, the results support the integration of behavioral regulation strategies, such as Positive Behavior Support (PBS), to mitigate externalizing and internalizing behaviors. Educators should be trained to use evidence-based classroom management techniques that reinforce prosocial behavior, while providing individualized support for children with self-regulation difficulties.

The predictive value of social skills and the level of early signs of LDs in regard to problem behaviors also highlight the need for early identification and tiered intervention systems. Schools and early childhood centers should adopt multi-tiered systems of support (MTSS) to provide differentiated services based on risk levels. For instance, children exhibiting a moderate-to-high level of early signs of LDs may benefit from small group interventions, led by school psychologists or special education teachers.

Given that early signs of LDs can evolve over time, the cross-sectional design of this study limits developmental interpretation. It is recommended that future research employ a longitudinal design to examine how early social–emotional signs predict later academic or behavioral outcomes.

Finally, these findings suggest that educational policy should support universal developmental screening and teacher training in regard to recognizing early behavioral and social–emotional difficulties. Equipping early educators with the knowledge and tools to detect and respond to these challenges can improve long-term academic and social outcomes for children at risk. By acting on these implications, educational practice can become more inclusive and responsive to the complex needs of children exhibiting early signs of LDs, ultimately fostering environments that support their holistic development and school readiness.

## Figures and Tables

**Table 1:** Descriptive findings regarding the difference between children’s problem behaviors and total social skills scores according to the level of early signs of LDs.

	Level of LD Risk	N	X	SS
Social Communication	Moderate	16	23.8	4.82
	Mild	115	27.0	4.59
	Very Low	22	30.5	7.82
Social Interaction	Moderate	16	23.8	4.98
	Mild	115	26.6	3.88
	Very Low	22	28.8	7.41
Social Independence	Moderate	16	22.3	4.91
	Mild	115	25.7	3.85
	Very Low	22	27.6	7.04
Total Social Skills Score	Moderate	16	69.9	11.94
	Mild	115	79.3	10.54
	Very Low	22	86.9	21.84
Externalizing Behaviors	Moderate	16	33.0	9.98
	Mild	115	27.8	11.78
	Very Low	22	18.2	11.17
Internalizing Behaviors	Moderate	16	19.5	6.37
	Mild	115	15.5	5.69
	Very Low	22	10.9	7.31
Total Problem Behavior Score	Moderate	16	52.5	14.79
	Mild	115	43.3	16.12
	Very Low	22	29.1	17.21

**Table 2:** One-way analysis of variance (Welch’s) results regarding the difference between children’s problem behavior and total social skills scores according to the level of early signs of LDs.

		Sum of Squares	df	Mean Square	F	Sig.	Eta Square
Social Communication	Between Groups	426.31	2	213.16	7.93	0.001 *	0.185
	Within Groups	4031.79	150	26.87			
	Total	4458.11	152				
Social Interaction	Between Groups	228.20	2	114.10	5.27	<0.006 *	0.146
	Within Groups	3246.26	150	21.64			
	Total	3474.47	152				
Social Independence	Between Groups	260.98	2	130.49	6.33	0.002 *	0.162
	Within Groups	3089.48	150	20.59			
	Total	3350.47	152				
Total Social Skills Score	Between Groups	2659.18	2	1329.59	8.03	0.001 *	0.186
	Within Groups	24,835.87	150	165.57			
	Total	27,495.05	152				
Externalizing Behaviors	Between Groups	2356.74	2	1178.37	8.86	0.001 *	0.197
	Within Groups	19,928.83	150	132.85			
	Total	22,285.58	152				
Internalizing Behaviors	Between Groups	710.97	2	355.48	9.82	0.001 *	0.209
	Within Groups	5426.56	150	36.17			
	Total	6137.54	152				
Total Problem Behavior Score	Between Groups	5613.95	2	2806.97	10.76	0.001 *	0.220
	Within Groups	39,109.34	150	260.72			
	Total	44,723.30	152				

* *p* < 0.007 (Bonferroni adjustment: 0.05/7 = 0.007).

**Table 3:** Problem behavior scale post hoc (Games–Howell post hoc test) results.

Occurrence of Early Signs of LDs		Moderate	Mild	Very Low
Problem Behavior Total (Games–Howell Post Hoc Test)				
Moderate	Mean difference	-	9.21	23.4
	*p*-value	-	0.077	0.001 *
Mild	Mean difference		-	14.2
	*p*-value		-	0.003 *
Very Low	Mean difference			-
	*p*-value			-
Internalizing Behaviors Total (Games–Howell Post Hoc Test)				
Moderate	Mean difference	-	4.00	8.59
	*p*-value	-	0.069	0.001 *
Mild	Mean difference		-	4.60
	*p*-value		-	0.025 *
Very Low	Mean difference			-
	*p*-value			-
Externalizing Behaviors Total (Games–Howell Post Hoc Test)				
Moderate	Mean difference	-	5.22	14.82
	*p*-value	-	0.159	0.001 *
Mild	Mean difference		-	9.60
	*p*-value		-	0.003 *
Very Low	Mean difference			-
	*p*-value			-

* *p* < 0.05.

**Table 4:** Social skills scale post hoc (Games–Howell post hoc test) results.

Occurrence of Early Signs of LDS		Moderate	Mild	Very Low
Social Skills Total (Games–Howell Post Hoc Test)				
Moderate	Mean difference	-	−9.37	−16.93
	*p*-value	-	0.021 *	0.012 *
Mild	Mean difference		-	−7.56
	*p*-value		-	0.271
Very Low	Mean difference			-
	*p*-value			-
Social Independence Total (Games–Howell Post Hoc Test)				
Moderate	Mean difference	-	−3.37	−5.28
	*p*-value	-	0.042 *	0.026 *
Mild	Mean difference		-	−1.90
	*p*-value		-	0.445
Very Low	Mean difference			-
	*p*-value			-
Social Interaction Total (Games–Howell Post Hoc Test)				
Moderate	Mean difference	-	−2.77	−4.96
	*p*-value	-	0.111	0.048 *
Mild	Mean difference		-	−2.19
	*p*-value		-	0.382
Very Low	Mean difference			-
	*p*-value			-
Social Communication Total (Games–Howell Post Hoc Test)				
Moderate	Mean difference	-	−3.22	−6.69
	*p*-value	-	0.052	0.007 *
Mild	Mean difference		-	−3.47
	*p*-value		-	0.131
Very Low	Mean difference			-
	*p*-value			-

* *p* < 0.05.

**Table 5:** Hierarchical regression analysis results.

Model	R^2^	ΔR^2^	F Change	Sig. F Change
1	0.14	0.14	23.79	0.000 *
2	0.19	0.05	17.31	0.002 *
3	0.19	0.00	0.62	0.536

* *p* < 0.001.

## Data Availability

The data presented in this study are available on request from the corresponding author. The data are not publicly available due to privacy or ethical restrictions approved by Anadolu University.
