# Correction: Aydin and Diken (2025). Examining Problem Behaviors and Social Skills in Preschoolers Exhibiting Early Signs of Learning Disabilities. *Behavioral Sciences*, *15*(8), 1087

**DOI:** 10.3390/bs15121616

**Published:** 2025-11-24

**Authors:** Yalcin Aydin, Ibrahim Halil Diken

**Affiliations:** 1Akademi Disleksi, 16230 Nilüfer, Türkiye; yalcinaydin@akademidisleksi.com; 2Research Institute for Individuals with Developmental Disabilities, Anadolu University, 26470 Tepebaşı, Türkiye

An error was identified in the original publication (Aydin & Diken, 2025) concerning the formatting of the references. While adapting the citations to match the journal’s style, an artificial intelligence tool was employed that, beyond reformatting, unintentionally altered some details. These included the replacement of journal titles, changes to volume and issue numbers, and in certain cases, the creation of references that did not exist. These issues went unnoticed at the time of submission.

As part of the correction, the entire reference list was thoroughly reviewed, all errors were amended, and DOIs were added where applicable to ensure the list’s accuracy and completeness. Additionally, the text was corrected to reflect the details in the reference list. This was carried out in addition to wording revisions.

## Missing Citation

On page 12, Section 4. Discussion, at the end of the second paragraph, “Kavale & Forness, 1996” was added.

On page 12, Section 4. Discussion, at the end of the fourth paragraph, “Kavale & Forness, 1996” was added.

On page 13, Section 4. Discussion, at the third paragraph, in lines 10–11, “Kılıç & Güngör Aytar, 2017” was added.

## Deleting Citation

On page 12, Section 4. Discussion, at the end of the second paragraph, “Clauser, 2015” and “Sucuoğlu & Demir, 2017” were deleted.

On page 12, Section 4. Discussion, at the end of the fourth paragraph, “Clauser, 2015”, “Sarı, 2011”, and “Mukaddes et al., 2014” were deleted.

On page 13, Section 4. Discussion, at the second paragraph, in line 9, “Pekdoğan, 2016” and “Bozkurt Polat et al., 2020” were deleted.

On page 13, Section 4. Discussion, at the end of the second paragraph, “Sapsağlam, 2015”, “Göktürk İnce, 2014”, and “Durualp & Aral, 2010” were deleted.

On page 13, Section 4. Discussion, at the third paragraph, in lines 10–11, “Bozkurt Polat et al., 2020” and “Girli, 2013” were deleted.

On page 13, Section 4. Discussion, at the end of the third paragraph, “Kuyurtar, 2011”, “Günindi, 2010”, and “Uysal & Balkan, 2015” were deleted.

## Text Correction

There were several errors in the original publication that have been corrected as follows.

On page 2, Section 1. Introduction, in the first paragraph, “(Ladd et al., 1999)” was corrected to “(Ladd et al., 1996)”.

On page 2, Section 1. Introduction, in the second paragraph, “Fantuzzo et al. (2003)” was corrected to “Fantuzzo et al. (2005)”.

On page 4, Section 2. Method, Subsection 2.3 Data Collection Tools, in the third paragraph, “(SSESLD; Okur & Aksoy, 2019)” was corrected to “(SSESLD; Okur & Aksoy, 2024)”.

On page 7, Section 3. Results, Subsection 3.1. Results Related to Levels of Early Signs of LDs, Problem Behaviors, and Social Skills Levels of Participant Children, in line 1, “assesses” was corrected to “assess”.

On page 12, Section 4. Discussion, in the second paragraph, “Elksnin (2004)” was corrected to “Elksnin and Elksnin (2004)”.

On page 12, Section 4. Discussion, in the second paragraph, “Webber & Scheruermann, 2008” was corrected to “Webber & Plotts, 2008”.

On page 12, Section 4. Discussion, in the third paragraph, the sentence “Sarı (2011) also found significant cognitive differences between children with and without learning disabilities, with the latter scoring considerably higher.” was deleted.

On page 12, Section 4. Discussion, in the fourth paragraph, “Webber & Scheruermann, 2008” was corrected to “Webber & Plotts, 2008”.

On page 13, Section 4. Discussion, in the second paragraph, “Ekinci Vural & Gürşimşek, 2009” was corrected to “Vural, 2009”.

On page 13, Section 4. Discussion, in the second paragraph, “Ezmeci, 2019” was corrected to “Ezmeci et al., 2022”.

On page 13, Section 4.

Discussion, in the third paragraph, “Ezmeci, 2019” was corrected to “Ezmeci et al., 2022”.

On page 13, Section 4. Discussion, in the third paragraph, “Most et al., 2012” was corrected to “Most & Greenbank, 2000”.

On page 13, Section 4. Discussion, in the third paragraph, “Koglin & Petermann, 2011” was corrected to “Duering & Petermann, 2011”.

On page 14, Section 4. Discussion, Subsection 4.2. Implications for Practice, in the first paragraph, “Booth-LaForce & Oxford, 2012” was corrected to “Booth-LaForce & Oxford, 2008”.

## References

All corrected references are listed here.

(Agaliotis & Kalyva, 2008). Agaliotis, I., & Kalyva, E. (2008). Nonverbal social interaction skills of children with learning disabilities. *Research in Developmental Disabilities*, *29*(1), 1–10. https://doi.org/10.1016/j.ridd.2006.09.002.

(Aksakal, 2020). Aksakal, B. (2020). *Oyun temelli sosyal beceri geliştirme programının koruyucu bakım altındaki 6 yaş çocukların sosyal beceri ve duygu düzenleme düzeyine etkisi [The effect of a play-based social skills development program on the social skills and emotion regulation levels of 6-year-old children under protective care]* [Master’s thesis, Bahçeşehir University].

(Aksoy & Baran, 2020). Aksoy, P., & Baran, G. (2020). Hikâye anlatma temelli ve oyun temelli sosyal beceri eğitiminin anasınıfı çocuklarının sosyal becerilerine etkisi: Deneysel bir çalışma [The effect of storytelling-based and play-based social skills training on the social skills of kindergarten children: An experimental study]. *Eğitim ve Bilim Dergisi [Education and Science]*, *45*(204), 157–183. https://doi.org/10.15390/EB.2020.8670.

(Aykır & Çiftçi-Tekinarslan, 2012). Aykır, T., & Çiftçi-Tekinarslan, İ. (2012). Okul öncesi dönemdeki zihinsel yetersizliği olan ve olmayan çocukların sosyal becerileri ve problem davranışlarının karşılaştırılması [Comparison of social skills and problem behaviors of preschool children with and without intellectual disabilities]. *Kastamonu Eğitim Dergisi [Kastamonu Education Journal]*, *20*(2), 627–648.

(Bloom & Heath, 2010). Bloom, E., & Heath, N. (2010). Recognition, expression, and understanding facial expressions of emotion in adolescents with nonverbal and general learning disabilities. *Journal of Learning Disabilities*, *43*, 180–192. https://doi.org/10.1177/0022219409345014.

(Booth-LaForce & Oxford, 2008). Booth-LaForce, C., & Oxford, M. L. (2008). Trajectories of social withdrawal from grades 1 to 6: Prediction from early parenting, attachment, and temperament. *Developmental Psychology*, *44*(5), 1298–1313. https://doi.org/10.1037/a0012954.

(Bull & Espy, 2006). Bull, R., & Espy, K. A. (2006). Working memory, executive functioning, and children’s mathematics performance. *Developmental Neuropsychology*, *30*, 685–699.

(Çayır & Eid, 2010). Çayır, A., & Eid, B. N. K. (2010). Öğrenme güçlüğü çeken bir ilköğretim 3. sınıf öğrencisinin kaynaştırma sınıfındaki sosyal uyum becerilerinin incelenmesi [An investigation of the social adaptation skills of a third-grade primary school student with learning difficulties in an inclusive classroom]. *Education Sciences*, *5*(4), 1764–1776.

(Cen & Aytaç, 2017). Cen, S., & Aytac, B. (2017). Ecocultural perspective in learning disability: Family support resources, values, child problem behaviors. *Learning Disability Quarterly*, *40*(2), 114–127. https://doi.org/10.1177/0731948716683516.

(Ceylan & Yiğitalp, 2018). Ceylan, R., & Yiğitalp, N. (2018). Aile katılımlı ve aile katılımsız sosyal beceri eğitiminin çocukların sosyal becerilerine etkisi [The effect of social skills training with and without parental involvement on children’s social skills]. *Anemon Muş Alparslan Üniversitesi Sosyal Bilimler Dergisi [Anemon Muş Alparslan University Journal of Social Sciences]*, *6*(6), 1119–1127. https://doi.org/10.18506/anemon.453737.

(Değirmenci, 2022). Değirmenci, G. Y. (2022). *Sosyal beceri eğitimi programının ÖG olan çocukların sosyal becerileri üzerindeki etkililiğinin incelenmesi [The investigation of the effectiveness of a social skills training program on the social skills of children with learning disabilities]* [Doctoral dissertation, Hacettepe University].

(Duering & Petermann, 2011). Duering, U. E., & Petermann, F. (2011). The effectiveness of the behavioural training for preschool children. *European Early Childhood Education Research Journal*, *19*(1), 97–111.

(Elksnin & Elksnin, 2004). Elksnin, L. K., & Elksnin, N. (2004). The social—Emotional side of learning disabilities. *Learning Disabilities Quarterly*, *27*(1), 3–8.

(Ezmeci et al., 2022). Ezmeci, F., Parpucu, N., & Akman, B. (2022). The mediating role of self-regulation skills in the relationship between social skills and problem behaviors in the early childhood period. *Psycho-Educational Research Reviews*, *11*(3), 738–750. https://doi.org/10.52963/PERR_Biruni_V11.N3.06.

(Fantuzzo et al., 2005). Fantuzzo, J. W., Bulotsky-Shearer, R., Fuscoa, R. A., & McWayne, C. (2005). An investigation of preschool classroom behavioral adjustment problems and social–emotional school readiness competencies. *Early Childhood Research Quarterly*, *20*, 259–275.

(Fazlıoğlu et al., 2011). Fazlıoğlu, Y., Okyay, L., & Ilgaz, G. (2011). Okulöncesi ve Anaokulu Davranış Ölçeğinin geçerlik ve güvenirlik çalışması [Validity and reliability study of the Preschool and Kindergarten Behavior Scale]. *Trakya Üniversitesi Sosyal Bilimler Dergisi [Trakya University Journal of Social Sciences]*, *13*(1), 242–254.

(Gresham et al., 2001) Gresham, F. M., Lane, K. L., & Lambros, K. M. (2001). Comorbidity of conduct problems and ADHD: Identification of “fledgling psychopaths”. In H. M. Walker, & M. H. Epstein (Eds.), *Making schools safer and violence free: Critical issues, solutions, and recommended practices* (pp. 17–27). PRO-ED.

(Heiervang et al., 2001) Heiervang, E., Stevenson, J., Lund, A., & Hugdahl, K. (2001). Behaviour problems in children with dyslexia. *Nordic Journal of Psychiatry*, *55*(4), 251–256. https://doi.org/10.1080/080394801681019101.

(Kavale & Forness, 1996) Kavale, K. A., & Forness, S. R. (1996). Social skill deficits and learning disabilities: A meta-analysis. *Journal of Learning Disabilities*, *29*(3), 226–237. https://doi.org/10.1177/002221949602900301.

(Kılıç & Güngör Aytar, 2017). Kılıç, K., & Güngör Aytar, F. A. (2017). The effect of social skills training on social skills in early childhood, the relationship between social skills and temperament. *Education and Science*, *42*(191), 185–204. https://doi.org/10.15390/EB.2017.7162.

(Kılıç-Tülü & Ergül, 2016). Kılıç-Tülü, B., & Ergül, C. (2016). Öğrenme güçlüğü olan çocukların duyguları tanıma becerileri [Emotion recognition skills of children with learning disabilities]. *Ankara Üniversitesi Eğitim Bilimleri Fakültesi Özel Eğitim Dergisi [Ankara University Faculty of Educational Sciences Journal of Special Education]*, *17*(3), 207–229. https://doi.org/10.21565/ozelegitimdergisi.267314.

(Ladd et al., 1996). Ladd, G. W., Kochenderfer, B. J., & Coleman, C. C. (1996). Friendship quality as a predictor of young children’s early school adjustment. *Child Development*, *67*(3), 1103–1118.

(Manassis & Young, 2000). Manassis, K., & Young, A. (2000). Perception of emotions in anxious and learning disabled children. *Depression and Anxiety*, *12*(4), 209–216. https://doi.org/10.1002/1520-6394(2000)12:4<209::AID-DA4>3.0.CO;2-A.

(McCoy et al., 2012). McCoy, S., Banks, J., & Shevlin, M. (2012). School matters: How context influences the identification of different types of special educational needs. *Irish Educational Studies*, *2*(2), 119–138.

(Milligan et al., 2016). Milligan, K., Phillips, M., & Morgan, A. S. (2016). Tailoring social competence interventions for children with learning disabilities. *Journal of Child and Family Studies*, *25*, 856–869. https://doi.org/10.1007/s10826-015-0278-4.

(Most & Greenbank, 2000). Most, T., & Greenbank, A. (2000). Auditory, visual, and auditory-visual, perception of emotions by adolescents with and without learning disabilities, and their relationship to social skills. *Learning Disabilities Research & Practice*, *15*(4), 171–178.

(Okur & Aksoy, 2024). Okur, M., & Aksoy, V. (2024). Öğrenme güçlüğü erken belirtileri tarama ölçeğinin psikometrik niteliklerinin belirlenmesi [Determination of the psychometric properties of the early signs of learning difficulties screening scale]. *Journal of Uludag University Faculty of Education*, *37*(2), 534–554. https://doi.org/10.19171/uefad.1401110.

(Özbey, 2009). Özbey, S. (2009). *Anaokulu ve Anasınıfı Davranış Ölçeğinin geçerlik güvenirlik çalışması ve destekleyici eğitim programının etkisinin incelenmesi [Validity and reliability study of the Preschool and Kindergarten Behavior Scale and examination of the effect of the supportive education program]* [Doctoral dissertation, Gazi University].

(Özen, 2015). Özen, K. (2015). Özel öğrenme güçlüğü tanısı almış 7–9 yaş çocukların geliştirdikleri zihin kuramı yeteneklerinin sağlıklı gelişim gösteren grup ile karşılaştırılması [Comparison of theory of mind abilities developed by 7–9-year-old children diagnosed with specific learning disabilities and their typically developing peers]. *Hacettepe University Faculty of Health Sciences Journal*.

(Pijl et al., 2013). Pijl, S. J., Frostad, P., & Mjaavatn, P. E. (2013). Students with special educational needs in secondary education: Are they intending to learn or to leave. *European Journal of Special Needs Education*, *29*(1), 16–28.

(Raimundo et al., 2013). Raimundo, R., Marques-Pinto, A., & Lima, M. L. (2013). The effects of a social–emotional learning program on elementary school children: The role of pupils’ characteristics. *Psychology in Schools*, *50*(2), 165–180.

(Remington et al., 2007). Remington, B., Hastings, R. P., Kovshoff, H., Degli Espinosa, F., Jahr, E., Brown, T., & Ward, N. (2007). Early intensive behavioral intervention: Outcomes for children with autism and their parents after two years. *American Journal on Mental Retardation*, *112*(6), 418–438.

(Tokuşlu, 2022). Tokuşlu, H. (2022). *Oyun temelli sosyal uyum programının 5–6 yaş grubundaki çocukların sosyal becerileri ve oyun becerileri üzerindeki etkisinin incelenmesi [The investigation of the effect of a play-based social adaptation program on the social and play skills of children aged 5–6 years]* [Master’s thesis, Hacettepe University].

(Torgesen, 2000). Torgesen, J. K. (2000). Individual differences in response to early interventions in reading: The lingering problem of treatment resisters. *Learning Disabilities Research & Practice*, *15*(1), 55–64. https://doi.org/10.1207/SLDRP1501_6.

(Vural, 2009). Vural, D. E. (2009). Okul öncesi eğitimde aile katılımlı sosyal beceri eğitimi [Parental involvement in social skills education in preschool education]. *Education Sciences*, *4*(3), 1110–1122.

(Webber & Plotts, 2008). Webber, J., & Plotts, C. A. (2008). *Emotional and behavioral disorders: Theory and practice*. Pearson.

The authors state that the scientific conclusions are unaffected. This correction was approved by the Academic Editor. The original publication has also been updated.
